# Seroprevalence of Antibodies to SARS-CoV-2 in Guangdong Province, China between March to June 2020

**DOI:** 10.3390/pathogens10111505

**Published:** 2021-11-18

**Authors:** Cheng Xiao, Nancy Hiu Lan Leung, Yating Cheng, Hui Lei, Shiman Ling, Xia Lin, Ran Tao, Xianzhong Huang, Wenda Guan, Zifeng Yang, Benjamin John Cowling, Mark Zanin, Sook-San Wong

**Affiliations:** 1 State Key Laboratory of Respiratory Disease, National Clinical Research Center for Respiratory Disease, Guangzhou Institute of Respiratory Health, The First Affiliated Hospital of Guangzhou Medical University, Guangzhou Medical University, Guangzhou 510182, China; xiaocheng1004@stu.gzhmu.edu.cn (C.X.); 2019310641@stu.gzhmu.edu.cn (H.L.); 2019217860@stu.gzhmu.edu.cn (S.L.); linxia@stu.gzhmu.edu.cn (X.L.); guanwenda2004@163.com (W.G.); jeffyah@163.com (Z.Y.); mark.zanin@gird.cn (M.Z.); 2 WHO Collaborating Centre for Infectious Disease Epidemiology and Control, School of Public Health, Li Ka Shing Faculty of Medicine, The University of Hong Kong, Hong Kong, China; leungnan@hku.hk (N.H.L.L.); bcowling@hku.hk (B.J.C.); 3 Laboratory Diagnosis Department, Guangzhou Kingmed Center for Clinical Laboratory, International Biotech Island, Guangzhou 510182, China; labcyt@kingmed.com.cn (Y.C.); labtr@kingmed.com.cn (R.T.); labhxz@kingmed.com.cn (X.H.); 4 Department of Clinical Laboratory Medicine, Guangzhou Medical University, Guangzhou 510182, China; 5 Faculty of Chinese Medicine, Macau University of Science and Technology, Macau 519020, China

**Keywords:** SARS-CoV-2, coronavirus disease 2019, seroprevalence, Guangdong, China, antibody

## Abstract

Guangdong province, located in South China, is an important economic hub with a large domestic migrant population and was among the earliest areas to report COVID-19 cases outside of Wuhan. We conducted a cross-sectional, age-stratified serosurvey to determine the seroprevalence of antibodies against SARS-CoV-2 after the emergence of COVID-19 in Guangdong. We tested 14,629 residual serum samples that were submitted for clinical testing from 21 prefectures between March and June 2020 for SARS-CoV-2 antibodies using a magnetic particle based chemiluminescent enzyme immunoassay and validated the results using a pseudovirus neutralization assay. We found 21 samples positive for SARS-CoV-2 IgG, resulting in an estimated age- and sex-weighted seroprevalence of 0.15% (95% CI: 0.06–0.24%). The overall age-specific seroprevalence was 0.07% (95% CI: 0.01–0.24%) in persons up to 9 years old, 0.22% (95% CI: 0.03–0.79%) in persons aged 10–19, 0.16% (95% CI: 0.07–0.33%) in persons aged 20–39, 0.13% (95% CI: 0.03–0.33%) in persons aged 40–59 and 0.18% (95% CI: 0.07–0.40%) in persons ≥60 years old. Fourteen (67%) samples had pseudovirus neutralization titers to S-protein, suggesting most of the IgG-positive samples were true-positives. Seroprevalence of antibodies to SARS-CoV-2 was low, indicating that there were no hidden epidemics during this period. Vaccination is urgently needed to increase population immunity to SARS-CoV-2.

## 1. Introduction

Guangdong, located in South China, is the most populous province and an important economic hub of China. It has a population of 115 million and the largest economy in China [1]. The major economic and urban areas of Guangdong are centered around nine cities within the Pearl River Delta (PRD) that has a combined population of 63 million people. Due to its economic importance, 30% of its population consists of migrants from other provinces, making it the province with the most domestic migration in China [2]. Shenzhen and Guangzhou, both first-tier megalopolises in Guangdong, are major domestic and international transportation hubs and were among the earliest cities outside of Wuhan to report cases of COVID-19 [3,4]. Guangdong was also among the first provinces in China to declare a Level I public health emergency, the nation’s highest emergency response level, on 23 January (Table S1) [5].

Seroprevalence studies have been used to inform the extent of transmission (including subclinical infections), herd immunity and the effectiveness of case-based surveillance in the community [6,7,8]. Given the importance of Guangdong as an economic, domestic migration and international transport hub, we investigated the community-wide SARS-CoV-2 virus transmission in Guangdong after the emergence of the virus, by conducting a cross-sectional seroprevalence study across province. Our aim was to estimate the age-specific seroprevalence of antibodies to SARS-CoV-2 in Guangdong province after the first wave of COVID-19 in China.

## 2. Results

### 2.1. Reported COVID-19 Cases in Guangdong Province between 19 January and 1 July 2020

**Figure 1:**
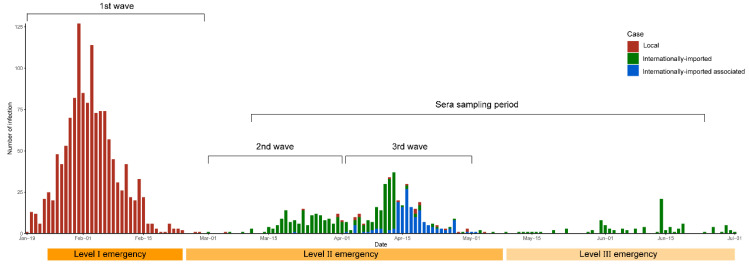
Cases of SARS-CoV-2 infections in Guangdong between 19 January and 1 July 2020. Case numbers represent the total of symptomatic and asymptomatic infections. (Local cases: Cases infected in Guangdong or imported from other provinces in China; Internationally imported cases: Individuals with SARS-CoV-2 infections returned from overseas; Internationally imported associated cases: Local cases identified as being associated with the internationally imported cases.).

**Figure 2:**
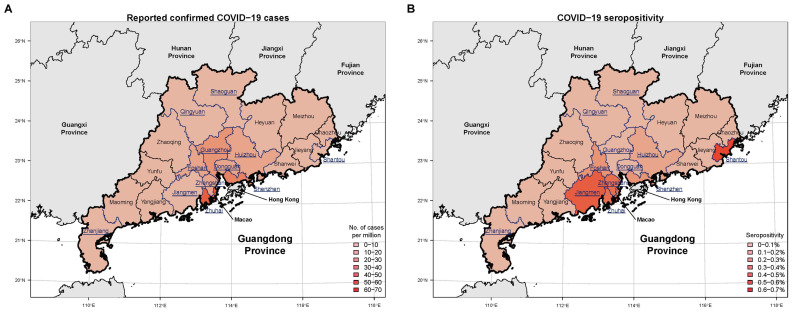
(**A**) Reported confirmed COVID-19 cases based on local official surveillance data in the different prefectural cities in Guangdong province. Asymptomatic infections were not available at a prefectural-city level. (**B**) Seropositivity of antibodies to SARS-CoV-2 as identified from the present study, in prefectural cities of the Guangdong province within the first six-months post COVID-19 emergence. Cities that had relatively higher connectivity with Wuhan prior to 23 January 2020, were underlined and highlighted in blue.

Between 19 January and 1 July 2020, Guangdong recorded 1961 laboratory-confirmed SARS-CoV-2 infections, with 1642 symptomatic COVID-19 cases, over three waves of activity that were characterized by distinct infection sources (Figure 1). The first wave, from January 14 to February 29, with a total of 1350 COVID-19 cases, was largely seeded by cases originating from Wuhan or Hubei [9] and remained the largest epidemic wave in the province so far. Cases were reported in 20 of the 21 prefecture-level cities in Guangdong (Yunfu reported no case) and were also highest in cities that had high migration activities from Wuhan in the five days prior to the national lockdown (Table 1, Figure 2A). The second outbreak, from March 1 to April 1, involved 161 infections that were mainly imported internationally while the third outbreak from 2 April to 2 May, involved 310 imported and associated-local cases. By 1 July, 1020 (62%) of the COVID-19 cases were reported mainly in Guangzhou and Shenzhen, which were also the major international port-of-entries, while the surrounding cities in the PRD recorded 478 (29%) cases (Table 1). As minimal cases were reported in all other cities after the first wave, the transmission trend across the province during the sera sampling period largely remained the same as in March.

### 2.2. Seroprevalence in Guangdong

Between 11 March and 24 June 2020, 14,629 sera were collected from 983 institutions across Guangdong. A total of 5264 (36%) and 9365 (64%) sera were collected from the low and high-risks cities, respectively. Large cities in high-risks area were generally better sampled while samples in the 10 to 19 years old group were generally under sampled, particularly in the-low risk cities (Table S2).

**Figure 3:**
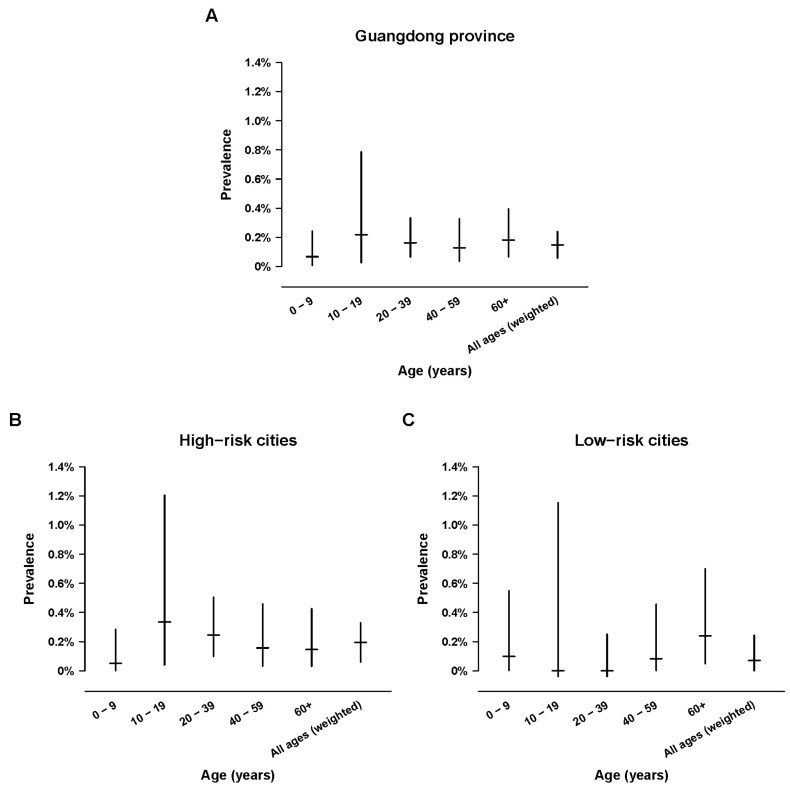
Estimates of age-specific SARS-CoV-2 seroprevalence in Guangdong province in (**A**) all cities or stratified by (**B**) high or (**C**) low risk of COVID-19 activities according to local official surveillance data. Seroprevalences in all ages were age- and sex-weighted according to the population structure of the included cities.

Out of 14,629 sera, we identified 21 (0.14%) samples positive for SARS-CoV-2 IgG by magnetic particle based chemiluminescent enzyme immunoassay (CLIA). We calculated that in Guangdong province overall, the estimated age and sex-weighted seroprevalence was 0.15% (95% CI 0.06% to 0.24%) (Figure 3A, Table S3). The weighted seroprevalence in high-risk cities was 0.19% (95% CI, 0.06% to 0.33%) (Figure 3B), approximately 2.7-fold higher than the weighted seroprevalence for low-risk cities of 0.07% (95% CI, 0% to 0.24%) (Figure 3C). In the whole of Guangdong, the lowest seroprevalence was detected in the youngest age-group ≤9 years old (0.07% (95%CI, 0.01% to 0.24%), while the seroprevalence estimates in the other age-groups were between 0.13% to 0.22% (Figure 3A). We noted apparent differences in the age-specific trends of seroprevalence estimates between the low and high risks region in Guangdong. In high-risk cities, age-specific seroprevalence was lowest in children ≤9 years of age, highest in adolescents, and lower in the three adult age groups (Figure 3B). In the low-risk cities, age-specific seroprevalence was higher in children 9 years old and older adults ≥40 years old, and lower in younger adults (Figure 3C).

In terms of geographical distribution, the seropositivity correlated with the number of reported COVID-19 cases in each city, with the notable exception of Guangzhou. Despite reporting the highest number of reported COVID-19 cases, it had the lowest seropositivity at 0.08% out of 2520 tested samples (Figure 2B, Table S2). This could be due to the high numbers of imported-associated cases that were detected during border screening and were subsequently quarantined at centralized facilities until determined to be PCR-negative, which effectively reduced the risk of virus spreading. In the low-risk region, seropositive samples were detected in Qingyuan (*n* = 1, 0.09%), Jiangmen (*n* = 1, 0.50%) and Shantou (*n* = 3, 0.60%). These three were amongst the low-risk cities in Guangdong that reported cases during the first wave and had high migrant connectivity with Wuhan (Table 1).

### 2.3. Proportion of Seropositive Samples with Neutralizing Titers

**Figure 4:**
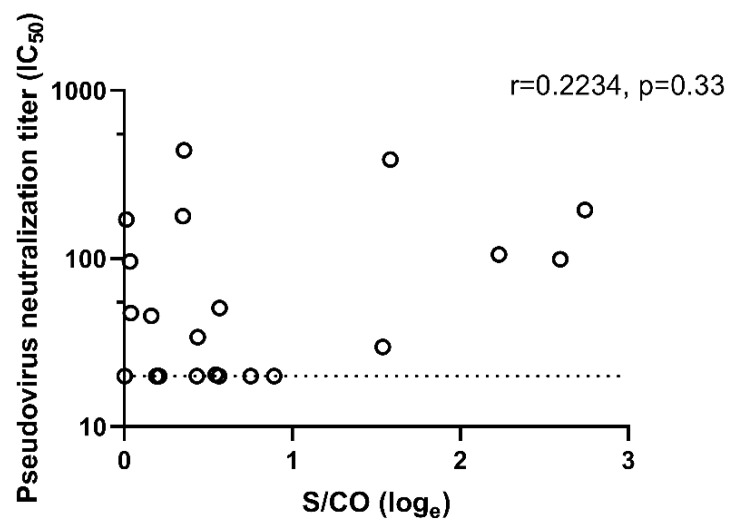
Correlation between the SARS-CoV-2 IgG titers and pseudovirus neutralization titers, expressed as 50% inhibitory concentration (IC_50_). The signal to cut-off (S/CO) readout on the X-axis were loge transformed to aid visualization of data points.

We tested the SARS-CoV-2 IgG positive samples for neutralization titers with the pseudovirus neutralization (pN) assay using a construct expressing the Spike (S) protein. Of the 21 samples, 14 (67%) had detectable neutralization activity at titers >20 (Table S4). The seven samples that did not have detectable neutralization activity had signal to cut-off readout (S/CO) that ranged from 1.002 to 2.442. There was no significant correlation between IgG-titer (expressed by the S/CO readout) with the measured IC_50_ titer (Pearson’s coefficient, r = 0.223, *p* = 0.33. Figure 4).

## 3. Discussion

In collaboration with a clinical testing laboratory, we were able to use residual serum samples that were submitted for clinical tests to conduct a cross-sectional serosurvey across the expanse of Guangdong province shortly after the emergence of COVID-19 in January 2020. Using a pseudovirus neutralization assay, we confirmed that 67% of the samples had neutralization titers, suggesting that most of the IgG-positive samples were true-positives. The remaining seven samples may still represent true positives as some infections may not have induced neutralizing antibodies, or their neutralizing antibodies may have waned to below the threshold of detection since being infected [10,11]. Studies have shown that the long-term antibody dynamics, particularly in those mild or asymptomatic COVID-19 cases can be variable [12,13]. The lack of correlation between the SAR-CoV-2 IgG-readout measured by CLIA with the pseudovirus neutralization titer could be due to the assay choices. The CLIA detects IgG against both S1 and N protein whereas our pseudovirus in the neutralization assay expressed only the S-protein and would therefore only account for neutralizing activity afforded by S-specific IgG.

Six months into the pandemic, the seroprevalence estimates based on residual sera collected from a clinical diagnostic laboratory network reported in our study were similar to other studies in the general community and lower than studies of high-risk individuals such as healthcare workers or hospital visitors in China (summarized in Table 2). Notably, two studies that included cohorts from Guangdong reported higher seroprevalence rates than ours. For example, by April 2020, Xu et al. found a seroprevalence of about 4% in healthcare workers or their exposed contacts in Wuhan, compared to approximately 1% in healthcare workers or factory workers in Guangzhou [14]. Separately, Liang et al. reported a seroprevalence of 2.1% and 0.6% amongst the 16,000 hospital patients and visitors in Wuhan and Guangzhou between January 25 to April 30 [15]. Collectively, these studies confirmed that SARS-CoV-2 virus transmission in other areas were minimal compared to Wuhan, but they did not include any validation tests to confirm the antibody specificity and provided limited information with regard to age-specific seroprevalence.

Our seroprevalence estimate is in line with the results of a large-scale nucleic acid testing conducted by Guangdong Centers for Disease Control and Prevention, which reported low number of PCR-positivity [9,19]. Between January 30 to March 19, they reported 1388 PCR-positive cases out of 1.6 million samples tested (0.089% positivity) [9]. In a follow-up study for the period up to July 9, only 385 samples in over 3.2 million samples tested by third-party institutions, were found to be positive (0.012% positivity) [19]. Notably, they observed changing age trends amongst the positive cases over time. In contrast to the first COVID-19 wave, during which the elderly (≥60 years old) comprised a significant proportion of infected cases (22.2%), PCR-positivity rates in the elderly declined during the subsequent waves (1.9%) but increased in the younger demographic, particularly in those between 20 to 39 years old (59.4%, which increased from 34.4% during the first wave). This was consistent with our data as we did not observe a higher seroprevalence among adults ≥60 years old. This trend was in contrast to those observed in Hubei [16], where evidence of infection was more common in those ≥60 years old. This suggests that cases in Guangdong after the first wave were more likely amongst the young, mobile travelers, particularly in the high-risk cities.

Guangdong was the epicenter of the first SARS outbreak in 2004 and regularly experienced zoonotic avian influenza cases. Consequently, the province had established an efficient surveillance and response system to emerging pathogens. The rapid response in the province appeared to have been effective in controlling the spread and emergence of SARS-CoV-2 locally. However, our seroprevalence trends suggests that the younger and mobile population in the urban centers of Guangdong as well as smaller cities with high connectivity may be a transmission risk and should be monitored. In conclusion, our data suggests an extremely low seroprevalence across Guangdong.

### Limitations of the Study

There were several limitations in our study. One major limitation was that the original sampling design was aimed at evaluating the age-specific seroprevalence in Guangdong at a city-prefecture level. However, the overall low seropositivity precluded this and we instead derived the seroprevalence estimates according to region of epidemic activity. Another limitation was the sampling bias that occurred between urban centers and small cities. It was easier to sample in urban centers due to the larger number of medical institutions available. Sampling was also particularly difficult for persons 10–19 years old, which resulted in the smaller sampling size of younger age groups than that of other age groups in our study. One of the reasons might be that younger individuals were less likely to seek non-emergency medical attention, especially during the period when epidemic control measures were in place. This resulted in wider confidence interval for the estimate of seroprevalence in this age group. Females were slightly oversampled compared to males (Figure S1), and therefore we also weighted by sex in addition to age to provide a more representative estimate. A meta-analysis of >3 millions COVID-19 global cases suggested there is no major difference in the risk of infection between sex [20], and therefore we expect that there would be minimal effect on the overall weighted estimate of seroprevalence due to the oversampling. In addition, our sampling period coincided with the gradual resumption of economic activity within the province as well as the easing of interprovincial travel restrictions. By April 8, the lockdown on Wuhan was lifted, signifying the last major travel restriction within China. As the cities with seropositive samples, including the three cities in the low-risks region, also had high numbers of travelers going to Wuhan in January, we are unable to determine if the positive samples were from a local resident or a returning migrant from Wuhan, or any other province. We also did not conduct IgM-testing, as we were interested in exposure history, for which IgG-antibody titers are more reliable [16]. Finally, as no data were available on asymptomatic infections prior to 1 April 2020 the number of infections in the early days that were reported will likely be underestimated.

## 4. Materials and Methods

### 4.1. Epidemiologic Data Source

Since 20 January 2020, COVID-19 has been designated as a notifiable infectious disease in China. Confirmed COVID-19 cases were defined based on the China’s National Health Council guideline issued at time of reporting [21,22,23,24,25] and were updated five times between 22 January and 7 March 2020 to keep up with the latest epidemiological and clinical developments [26]. A specific category for asymptomatic cases, which are cases with positive nucleic acid test but without clinical symptoms, were introduced after January 28, but these data were only publicly available after April 1. During the time of sampling for this study, definitions were based on the 6th edition, which was defined as persons with fever or respiratory symptoms and who had etiological, PCR- or serological evidence of infection (File S1).

Data of confirmed COVID-19 and asymptomatic cases were obtained from the Guangdong Provincial Health Authority (GPHA) (File S2) [27]. Number of asymptomatic cases were only reported at the province level (Figure 1) but not at the city level (Table 1). Case data were presented for the following two time periods: between January 19 to March 3, representing the first wave of COVID-19 in China; and time of study conception and up to July 1, coinciding with the week upon completion of sera sampling. For ease of description, we will use the term “infections” to mean symptomatic and asymptomatic SARS-CoV-2 infections in our manuscript.

Population size of each prefecture were sourced from the provincial statistics website and were based on the 2018 population numbers. However, to calculate the age- and sex-adjusted seroprevalence, the prefecture-city population structure were sourced from the 2015 Guangdong One-Percent Population Sample Survey [28], the most recent of such data that was available. As the period before the Wuhan lockdown coincided with the 2020 Spring Festival migration period, when migrants usually return home, we used the migration index sourced from *Baidu Qianxi* (Available online: https://qianxi.baidu.com/ (accessed on 1 May 2020)) in the five-days before 23 January, as an indicator of the degree of connectivity between Wuhan and cities in Guangdong. We selected the window of a 5-day period before the lockdown (on January 23) in our study as this captured the peak (which occurred on January 23) of the inflow/outflow migration from Hubei and Guangdong. The migration index represents the percentage of the daily number of inbound and outbound events by rail, air and road traffic (provided as File S3).

### 4.2. Study Design

This study was conducted in collaboration with Kingmed Diagnostic Laboratory Services, which provide services across a large network of hospital and medical institutions in China. We collected a total of 14,629 serum samples that remained after being used in the original clinical tests (residual serum) in the following age groups: 0–9, 10–19, 20–39, 40–59 and ≥60 years old. The serum samples, originally collected in 1 to 2 mL standard serum separator tubes, were submitted within 24 h to Kingmed’s central laboratory in Guangzhou from all 21 prefecture-level cities in Guangdong province between 11 March and 24 June 2020. After the original clinical tests were done, the residual sera were stored at −20 °C until use in our study. We determined that at least 300 sera samples collected per group would allow the estimation of age-specific seroprevalence within ±1.7%, assuming a minimal level of 0.1% age-specific seroprevalence following a binomial distribution. We classified the prefectural cities in Guangdong as low-risk or high-risk to represent regions that experienced low and high COVID-19 activity in the province based on the GPHA surveillance data. Low-risks were cities that reported less than 10 confirmed COVID-19 cases per million population, while high-risks were cities that reported 10 or more cases per million population by 3 March 2020. We selected sera submitted for blood chemistry tests but excluded those that were submitted for autoimmune or cancer screening. Information about age, sex, source of serum samples and date of sample collection were also retrieved.

### 4.3. Serologic Assays

We utilized a tiered-testing system to identify SARS-CoV-2 IgG and neutralizing antibodies as an indicator of past exposure to SARS-CoV-2 infection. We first used a commercially available magnetic particle-based chemiluminescent enzyme immunoassay (CLIA) that detects IgG to two SARS-CoV-2 antigens in a single reaction; a peptide to the Spike (S) and the Nucleocapsid (NP) proteins (Bioscience, China). All samples were inactivated at 56 °C for 30 minutes and tested according to the manufacturer’s instructions. S/CO ratio > 1.0 was considered positive. Samples were considered to be SARS-CoV-2 IgG-positive if results from two rounds of testing both yielded S/CO > 1.0. The overall assay specificity and sensitivity was 99.2% and 89.6% based on testing of 282 sera from individuals with laboratory-confirmed COVID-19. However, the assay’s sensitivity increases to 100% in samples that were collected 17 days after symptom onset [29].

Samples determined to be positive using the CLIA assay were then tested using a lentivirus-based-pseudovirus expressing the SARS-CoV-2 Spike protein neutralization assay as previously described [30].

### 4.4. Statistical Analysis

We estimated crude age-specific COVID-19 seroprevalences and the confidence intervals in five age-groups (0–9, 10–19, 20–39, 40–59 and ≥60 years) for each prefecture city, the low- and high-risk cities, or Guangdong province using binomial approximation. We also estimated the age- and sex-weighted seroprevalences for low-risk, high-risk cities and Guangdong province using data on the population structure of Guangdong province based on the 2015 Guangdong One-Percent Population Sample Survey. The estimations were conducted with R version 3.6.3. For the neutralization titer, the IC_50_ was calculated using the three-parameter non-linear regression function in Prism 8.0 (GraphPad). The correlation between S/CO readout and neutralization titer were determined using Pearson’s Correlation Test, also in Prism 8.0. The Pearson’s correlation test was performed under the assumption that the data was sampled from a Gaussian distribution, with the *p*-value derived from a two-tailed test.

## Figures and Tables

**Table 1:** Demographic of the twenty-one prefecture-cities in Guangdong; number of confirmed COVID-19 cases between January 19 to March 3, representing the first COVID-19 wave in Guangdong; and between January 19 to July 1, representing the first 6-months post COVID-19 emergence in Guangdong.

Prefecture	Total Cases by March 3 ^a^ *n*(%)	Total Cases by July 1 ^a^ *n*(%)	Population (Million) ^b^	Female (%) ^a^	Incidence (Per Million)	Designated Risk Level	Migration Index with Wuhan ^c^
Yunfu	0 (0)	0 (0)	2.5269	49	0	Low	0
Heyuan	4 (0.3)	5 (0.3)	3.0939	49	1.29	Low	0
Jieyang	8 (0.6)	11 (0.7)	6.0894	49	1.32	Low	0
Shanwei	5 (0.4)	5 (0.3)	2.9936	47	1.67	Low	0
Chaozhou	5 (0.4)	6 (0.4)	2.6566	50	1.81	Low	0
Maoming	14 (1.0)	14 (0.9)	6.3132	47	2.22	Low	0
Meizhou	16 (1.2)	17 (1.0)	4.3788	51	2.91	Low	0
Zhanjiang	22 (1.6)	24 (1.5)	7.3320	47	3.00	Low	1,2
Qingyuan	12 (0.9)	12 (0.7)	3.8740	49	3.10	Low	2
Shaoguan	10 (0.7)	10 (0.6)	2.9976	50	3.34	Low	2
Shantou	25 (1.9)	26 (1.6)	5.6385	50	4.44	Low	2
Zhaoqing *	19 (1.4)	20 (1.2)	4.1517	49	4.58	Low	0
Jiangmen *	23 (1.7)	24 (1.5)	4.5982	49	5.01	Low	2
Yangjiang	14 (1.0)	14 (0.9)	2.5556	47	5.51	Low	0
Foshan *	84 (6.2)	100 (6.1)	7.9057	46	10.63	High	2
Dongguan *	99 (7.3)	100 (6.1)	8.3922	44	11.80	High	1,2
Huizhou *	62 (4.6)	62 (3.8)	4.8300	47	12.84	High	1,2
Zhongshan *	66 (4.9)	69 (4.2)	3.3100	46	19.94	High	2
Guangzhou *	346 (25.6)	558 (33.9)	14.9044	49	23.22	High	1,2
Shenzhen *	418 (31.0)	462 (28.2)	13.0266	46	32.10	High	1,2
Zhuhai *	98 (7.3)	103 (6.3)	1.8911	48	51.85	High	1,2
Total (%)	1350 (100)	1642 (100)	113.46				
Average				48	9.65		

^a^ Does not include asymptomatic cases. ^b^ Source: Based on 2018 population data (Guangdong Provincial Bureau of Statistics). ^c^ 0 = low migration activity with Wuhan, 1 = high incoming migration from Wuhan, 2 = high outgoing migration to Wuhan. All data was for the period between 18 to 22 January 2020. * Prefectural cities in the Pearl River Delta.

**Table 2:** Summary of seroprevalence studies of SARS-CoV-2 antibodies in China within the first 6-months of COVID-19 emergence.

Author	Location	Sampling Period	Seropositivity for IgG or IgG and IgM (%)	95% Confidence Interval	Total Population Surveyed	Sampling Population	Approach
Xu et al. [14]	Wuhan	March–April 2020	3.8	2.6 to 5.4	714	Healthcare Workers	Screening
	Guangzhou, Foshan		2.80	1.8 to 4.6	563	Hemodialysis Patients	
			1.20	0.4 to 3.3	260	Healthcare Workers	
			1.40	0.6 to 2.9	442	Factory Workers	
	Sichuan		0.58	0.45 to 0.76	9442	General Community	
Liang et al. [15]	Wuhan	January–April 2020	2.10	Not reported	8272	Hospital Visitors	Residual Sera
	Guangzhou		0.60	Not reported	8782	Hospital Visitors	Residual Sera
Pan et al. [16]	Wuhan	May 2020	2.39	2.27 to 2.52	61,437	General Community	Cluster Sampling
Liu et al. [17]	Wuhan	February–April 2020	4.60	4.3 to 4.9	19,555	General Workers	Screening
Chang et al. [18]	Shijiazhuang	April 2020	0.0074	0.0013 to 0.042	13,540	Blood Donors	Residual Sera
	Shenzhen		0.029	0.0081 to 0.11	6810		
	Wuhan		2.29	2.08 to 2.52	17,794		

## Data Availability

The data available in this study are available on request from the corresponding author. The data are not publicly available due to ethical reasons.

## Supplementary Materials

The following are available online at https://www.mdpi.com/article/10.3390/pathogens10111505/s1, Table S1: Timeline and details of the public health response implemented after the emergence of COVID-19 in Guangdong province. Table S2: The seropositivity for antibodies to SARS-CoV-2 in the prefectural cities of Guangdong province identified in the present study between 11 March and 24 June 2020. Since the number of seropositive samples were too low to estimate seroprevalence at the individual city level, percentage of seropositivity were used instead. Table S3: The age-specific SARS-CoV-2 seroprevalence in Guangdong province, and for cities of low or high risk of COVID-19 activities according to the local official surveillance data. The crude seroprevalences for individual age groups were presented, while the overall age- and sex-weighted seroprevalences for cities of low or high risk of COVID-19 activities, and that of Guangdong province, were presented. Table S4: Pseudovirus neutralization titers, expressed as 50% inhibitory concentration (IC_50_) of the SARS-CoV-2 IgG-positive samples. Figure S1: The population structure in Guangdong Province and sample structure in this study. File S1: Case definition of COVID-19 according to the Protocol on Prevention and Control of Novel Coronavirus Pneumonia (Edition 2 to 7). File S2: Daily case numbers of COVID-19 in Guangdong between 19 January and 1 July 2020. The daily numbers of asymptomatic cases prior to 1 April 2020 were not publicly accessible. File S3: Daily percentage of inbound and outbound events by rail, air and road traffic to and from Wuhan in the five days prior to the lockdown. Only the daily top 100-destination cities were reported by the website. Cities in Guangdong that were listed in daily the top-100 lists were highlighted.
