# Supplementary material for: Seroprevalence of Antibodies to SARS-CoV-2 in Guangdong Province, China between March to June 2020
Source: Pathogens. 2021 Nov 18;10(11):1505. doi: 10.3390/pathogens10111505 (PMC8619097; doi:10.3390/pathogens10111505)
Supplement: Supplementary file 1 [file pathogens-10-01505-s001.zip › Supplemental_File_S1.pdf]

Supplemental File S1. Case definition of COVID-19 according to the Protocol on Prevention and Control of Novel Coronavirus Pneumonia (Edition 2 to 7).

Definition of COVID-19 cases according to the guideline released by China's National Health Council.

During the sampling time (March 11 to June 24 2020), the case definition were based on Edition 6.

1. Protocol on Prevention and Control of Novel Coronavirus Pneumonia (Edition 7)

Release Date: September 15th 2020
http://www.nhc.gov.cn/cms-search/xxgk/getManuscriptXxgk.htm?id=318683cbfaee4191aee29cd774b19d8d

Suspect cases

1) Had any one of the epidemiological history plus any two of the clinical manifestations;
2) If no epidemiological history, it needs to conform to any two of clinical manifestations plus SARS-CoV-2 IgM positive;
3) Conform to all three of clinical manifestations.

Epidemiological history

1) Had a travel or residence history of the community with confirmed cases or asymptomatic cases within 14 days prior to the onset of the disease;
2) In contact with novel coronavirus infected people (with positive results for the nucleic acid test) within 14 days prior to the onset of the disease;
3) Had an exposure to people with fever and/or respiratory symptoms and come from the community which had reported confirmed or asymptomatic cases.
4) Clustered cases (2 or more cases with fever and/or respiratory symptoms in a small area such families, offices, schools, workshops etc within 14 days).

Clinical manifestations

1) Fever and/or respiratory symptoms, etc.
2) Radiologic characteristics of COVID-19.
3) Normal or decreased WBC count, normal or decreased lymphocyte count in the early stage of onset.

Confirmed cases

Suspect cases with one of the following etiological or serological evidences:
(1) Real-time fluorescent RT-PCR indicates positive for new coronavirus nucleic acid;
(2) Viral gene sequence is highly homologous to known new coronaviruses.
(3) SARS-CoV-2 specific Ig M and IgG are detectable in serum; Specific IgG is detectable or reaches a titration of at least 4-fold increase during convalescence compared with the acute phase.

Asymptomatic infected persons

Respiratory specimens tested as SARS-CoV-2 positive in etiology, no relevant clinical manifestations, such as fever, cough, sore throat. No radiologic characteristics of COVID-19. Two types of asymptomatic infections: there were neither self-perception nor clinical recognition and signs of disease within 14 days of isolated medical observation, or it is in the incubation period of asymptomatic infection. If relevant symptoms or signs appear later, they should be revised to a confirmed case within 24 hours.

2. Protocol on Prevention and Control of Novel Coronavirus Pneumonia (Edition 6)

Release Date: March 7th 2020

http://www.nhc.gov.cn/jkj/s3577/202003/4856d5b0458141fa9f376853224d41d7.shtml

Suspect cases

1) A suspect case has any of the epidemiological history plus any two clinical manifestations, or
2) All three clinical manifestations if there is no clear epidemiological history.

(1) Epidemiological history

1) History of travel to or residence in Wuhan and its surrounding areas, or other communities in China where cases have been reported, or other countries/areas with severe outbreaks, within 14 days prior to the onset of the disease;

2) In contact with novel coronavirus infected people (with positive results for the nucleic acid test) within 14 days prior to the onset of the disease;

3) In contact with patients who have fever or respiratory symptoms from Wuhan and its surrounding area, or from communities where confirmed cases have been reported, or from other countries/areas with severe outbreaks, within 14 days before the onset of the disease; or

4) Clustered cases (2 or more cases with fever and/or respiratory symptoms in a small area such families, offices, schools, workshops etc within 14 days).

(2) Clinical manifestations

1) Fever and/or respiratory symptoms;

2) The aforementioned imaging characteristics of COVID-19;

3) Normal or decreased WBC count, normal or decreased lymphocyte count in the early stage of onset.

Confirmed cases

Suspect cases with one of the following etiological or serological evidences:

(1) Real-time fluorescent RT-PCR indicates positive for new coronavirus nucleic acid;

(2) Viral gene sequence is highly homologous to known new coronaviruses.

(3) SARS-CoV-2 specific Ig M and IgG are detectable in serum; SARS-CoV-2 specific IgG is detectable or reaches a titration of at least 4-fold increase during convalescence compared with the acute phase.

Asymptomatic infected persons

Asymptomatic people with SARS-CoV-2 positive in etiology in respiratory specimens or IgM detected in serum. They are mainly found through close contact tracing, investigation of clusters and infection source tracing. If relevant symptoms or signs appear later, they should be revised to a confirmed case timely.

3. Protocol on Prevention and Control of Novel Coronavirus Pneumonia (Edition 5)

Release Date: February 21st 2020

http://www.nhc.gov.cn/jkj/s3577/202002/a5d6f7b8c48c451c87dba14889b30147.shtml

Suspect cases

Same with Edition 6

Confirmed cases

Suspect cases with one of the following etiological or serological evidences:

(1) Real-time fluorescent RT-PCR indicates positive for new coronavirus nucleic acid;

(2) Viral gene sequence is highly homologous to known new coronaviruses.

Asymptomatic infected persons

Respiratory specimens tested as SARS-CoV-2 positive, no relevant clinical manifestations.

4. Protocol on Prevention and Control of Novel Coronavirus Pneumonia (Edition 4)

Release Date: February 7th 2020

http://www.nhc.gov.cn/jkj/s3577/202002/573340613ab243b3a7f61df260551dd4.shtml

The surveillance on suspect and confirmed cases was different between Hubei and outside Hubei.
The cases definition outside Hubei were consistent with V5.

5. Protocol on Prevention and Control of Novel Coronavirus Pneumonia (Edition 3)

Release Date: January 28th 2020

http://www.nhc.gov.cn/jkj/s7923/202001/470b128513fe46f086d79667db9f76a5.shtml

Suspect cases

1) A suspect case has any of the epidemiological history plus any two clinical manifestations, or
2) All three clinical manifestations if there is no clear epidemiological history.

<u>(1) Epidemiological history</u>

1) History of travel to or residence in Wuhan and its surrounding areas, or other communities in China where cases have been reported, or other countries/areas with severe outbreaks, within 14 days prior to the onset of the disease;
2) In contact with patients who have fever or respiratory symptoms from Wuhan and its surrounding area, or from communities where confirmed cases have been reported, or from other countries/areas with severe outbreaks, within 14 days before the onset of the disease; or
3) Clustered cases or related to confirmed cases.

<u>(2) Clinical manifestations</u>

1) Fever and/or respiratory symptoms;
2) The aforementioned imaging characteristics of COVID-19;
3) Normal or decreased WBC count, normal or decreased lymphocyte count in the early stage of onset.

Asymptomatic infected persons

Respiratory specimens tested as SARS-CoV-2 positive, no relevant clinical manifestations.

6. Protocol on Prevention and Control of Novel Coronavirus Pneumonia (Edition 2)

Release Date: January 22nd 2020

http://www.nhc.gov.cn/jkj/s3577/202001/c67cfe29ecf1470e8c7fc47d3b751e88.shtml

7. Protocol on Prevention and Control of Novel Coronavirus Pneumonia Edition 1 is not publicly available.
