# Supplementary material for: Seroprevalence of Antibodies to SARS-CoV-2 in Guangdong Province, China between March to June 2020
Source: Pathogens. 2021 Nov 18;10(11):1505. doi: 10.3390/pathogens10111505 (PMC8619097; doi:10.3390/pathogens10111505)
Supplement: Supplementary file 1 [file pathogens-10-01505-s001.zip › Supplemental_Table_S1.pdf]

Supplemental Table S1: Timeline and details of the public health response implemented after the emergence of COVID-19 in Guangdong.

**Level of Emergency**[a]		I	II	III
**Level of Response and authorization**		Extremely serious public health emergency. Coordinated at the State Council level	Serious public health emergency. Coordinated at the provincial level with guidance from the State Council (if required)	Public health emergency. Coordinated at the municipal level with guidance from the Provincial Health Commission (if required)
**Period**		January 23 to February 24, 2020	February 25 to May 8, 2020	May 9th to Present
Risk-based measures	Screening and isolation of fever cases	Yes	Yes	Yes
	Screening and isolation of residents in high risks communities	Yes	Yes	Yes
	Mandatory quarantine of close contacts and international travellers	Yes	Yes	Yes
Community-wide measures	Limited interprovincial/international travel	Yes	Yes	Yes
	Closure of schools, and businesses	Yes	Schools remained closed	Schools reopened in June
	Closure of public spaces	Yes	Gradual reopening of public spaces	Gradual reopening of public spaces
	Banning of public gatherings	Yes	Gradual easing of ban	No
	Banning of dine-in	Yes	Banned in high risks areas	No
	Mandatory masks in public areas	Yes	Yes	Yes
	Access restriction to private spaces	Yes	Yes	No
	Social distancing required	Yes	Recommended	No
	Temperature-scanning	Yes	Yes	Yes
	Usage of 'health codes'	Yes	Yes	Yes

[a] The national public health emergency consists of four-levels. Level IV response is the lowest level of emergency, whereby the response is coordinated at the district level with guidance from the Municipal Health Commission (if required). Guangdong is presently at Level III.
