# Supplementary material for: Seroprevalence of Antibodies to SARS-CoV-2 in Guangdong Province, China between March to June 2020
Source: Pathogens. 2021 Nov 18;10(11):1505. doi: 10.3390/pathogens10111505 (PMC8619097; doi:10.3390/pathogens10111505)
Supplement: Supplementary file 1 [file pathogens-10-01505-s001.zip › Supplemental_Table_S2.pdf]

Supplemental Table S2. The seropositivity for antibodies to SARS-CoV-2 in the prefectural cities of Guangdong province identified in the present study between March 11 and June 24, 2020. Since the number of seropositive samples were too low to estimate seroprevalence at the individual city level, percentage of seropositivity were used instead.

Perfecture-City	Risk Level	Population Size[a]	Total Tested	Percentage of population sampled (%)	No. Positive	Seropositivity(%)
Chaozhou	Low	2656600	195	0.007	0	0.000
Heyuan	Low	3093900	90	0.003	0	0.000
Jiangmen	Low	4598200	200	0.004	1	0.005
Jieyang	Low	6089400	435	0.007	0	0.000
Maoming	Low	6313200	886	0.014	0	0.000
Meizhou	Low	4378800	119	0.003	0	0.000
Qingyuan	Low	3874000	1126	0.029	1	0.089
Shantou	Low	5638500	497	0.009	3	0.604
Shanwei	Low	2993600	533	0.018	0	0.000
Shaoguan	Low	2997600	346	0.012	0	0.000
Yangjiang	Low	2555600	281	0.011	0	0.000
Yunfu	Low	2526900	161	0.006	0	0.000
Zhanjiang	Low	7332000	158	0.002	0	0.000
Zhaoqing	Low	4151700	237	0.006	0	0.000
Dongguan	High	8392200	1235	0.015	0	0.000
Foshan	High	7905700	1290	0.016	3	0.233
Guangzhou	High	14904400	2520	0.017	2	0.079
Huizhou	High	4830000	933	0.019	1	0.107
Shenzhen	High	13026600	2231	0.017	5	0.224
Zhongshan	High	3310000	450	0.014	2	0.444
Zhuhai	High	1891100	706	0.037	3	0.425
**Total**		**113460000**	**14629**		**21**	
**Average**				**0.013**		

[a] Source: Based on 2018 population data (to the nearest hundred) [29].
