# Supplementary material for: Seroprevalence of Antibodies to SARS-CoV-2 in Guangdong Province, China between March to June 2020
Source: Pathogens. 2021 Nov 18;10(11):1505. doi: 10.3390/pathogens10111505 (PMC8619097; doi:10.3390/pathogens10111505)
Supplement: Supplementary file 1 [file pathogens-10-01505-s001.zip › Supplemental_Table_S3.pdf]

Supplemental Table S3. The age-specific SARS-CoV-2 seroprevalence in Guangdong province, and for cities of low or high risk of COVID-19 activities according to the local official surveillance data. The crude seroprevalences for individual age groups were present, while the overall age- and sex-weighted seroprevalences for cities of low or high risk of COVID-19 activities, and that of Guangdong province, were present.

Risk level	Age Group	Population Size[a]	Total Tested	Percentage of population sampled (%)	No. Positive	Estimated Seroprevalence (%)	95% Confidence Interval (Lower)	95% Confidence Interval (Upper)
Low	0 to 9	8186351	1015	0.012	1	0.099	0.002	0.548
Low	10 to 19	8300244	318	0.004	0	0.000	0.000	1.153
Low	20 to 39	17121124	1464	0.009	0	0.000	0.000	0.252
Low	40 to 59	15915092	1218	0.008	1	0.082	0.002	0.457
Low	≥60	8787515	1249	0.014	3	0.240	0.050	0.700
**Low**	**All ages (weighted)**	**58310326**	**5264**	**0.009**	**5**	**0.071**	**0.000**	**0.244**
High	0 to 9	4882700	1954	0.040	1	0.051	0.001	0.285
High	10 to 19	4645156	597	0.013	2	0.335	0.041	1.205
High	20 to 39	22416946	2853	0.013	7	0.245	0.099	0.505
High	40 to 59	13736703	1910	0.014	3	0.157	0.032	0.458
High	≥60	4468732	2051	0.046	3	0.146	0.030	0.427
**High**	**All ages (weighted)**	**50150237**	**9365**	**0.019**	**16**	**0.194**	**0.060**	**0.329**
All	0 to 9	13069051	2969	0.023	2	0.067	0.008	0.243
All	10 to 19	12945400	915	0.007	2	0.219	0.026	0.787
All	20 to 39	39538070	4317	0.011	7	0.162	0.065	0.334
All	40 to 59	29651795	3128	0.011	4	0.128	0.035	0.327
All	≥60	13256247	3300	0.025	6	0.182	0.067	0.395
**All**	**All ages (weighted)**	**108460563**	**14629**	**0.013**	**21**	**0.148**	**0.057**	**0.240**

[a] Based on 2015 Guangdong One Percent Survey [21]
