# Supplementary material for: Seroprevalence of Antibodies to SARS-CoV-2 in Guangdong Province, China between March to June 2020
Source: Pathogens. 2021 Nov 18;10(11):1505. doi: 10.3390/pathogens10111505 (PMC8619097; doi:10.3390/pathogens10111505)
Supplement: Supplementary file 1 [file pathogens-10-01505-s001.zip › Supplemental_Table_S4.pdf]

Supplemental Table S4. Pseudoparticle neutralization titers, expressed as 50% inhibitory concentration (IC_{50}) of the SARS-CoV-2 IgG-positive samples, listed according to increasing S/CO values. S/CO refers to the signal/cut-off readout from the SARS-CoV-2 IgG-assay.

Sample	Age	Sex	Location	City Risk Level	S/CO[a]	IC_{50}[a]
**X1141**	60	Female	Foshan	High	1.002	<20
**X707**	31	Female	Shenzhen	High	1.012	171
**X178**	55	Male	Guangzhou	High	1.034	96
**X102**	21	Female	Huizhou	High	1.04	47
**X4898**	82	Male	Shantou	Low	1.174	46
**X89**	32	Female	Guangzhou	High	1.21	<20
**X162**	31	Female	Shenzhen	High	1.23	<20
**X12208**	50	Male	Qingyuan	Low	1.418	179
**X10986**	44	Female	Zhongshan	High	1.427	440
**X3727**	89	Male	Jiangmen	Low	1.541	<20
**X1475**	11	Male	Shenzhen	High	1.549	34
**X1337**	44	Female	Foshan	High	1.723	20
**X9951**	1	Female	Shantou	Low	1.759	<20
**X5032**	62	Male	Shantou	Low	1.764	51
**X11873**	71	Female	Zhuhai	High	2.121	<20
**X6922**	19	Female	Foshan	High	2.442	<20
**X510**	27	Female	Shenzhen	High	4.658	30
**X220**	30	Male	Zhongshan	High	4.878	388
**X3972**	4	Male	Shenzhen	High	9.3	106
**X12476**	76	Female	Zhuhai	High	13.406	99
**X9864**	36	Male	Zhuhai	High	15.515	194

[a] No significant correlation as determined using Pearson's correlation test (r=0.223, p=0.33)
