# Supplementary figures and images for: Seroprevalence of Antibodies to SARS-CoV-2 in Guangdong Province, China between March to June 2020
Source: Pathogens. 2021 Nov 18;10(11):1505. doi: 10.3390/pathogens10111505 (PMC8619097; doi:10.3390/pathogens10111505)

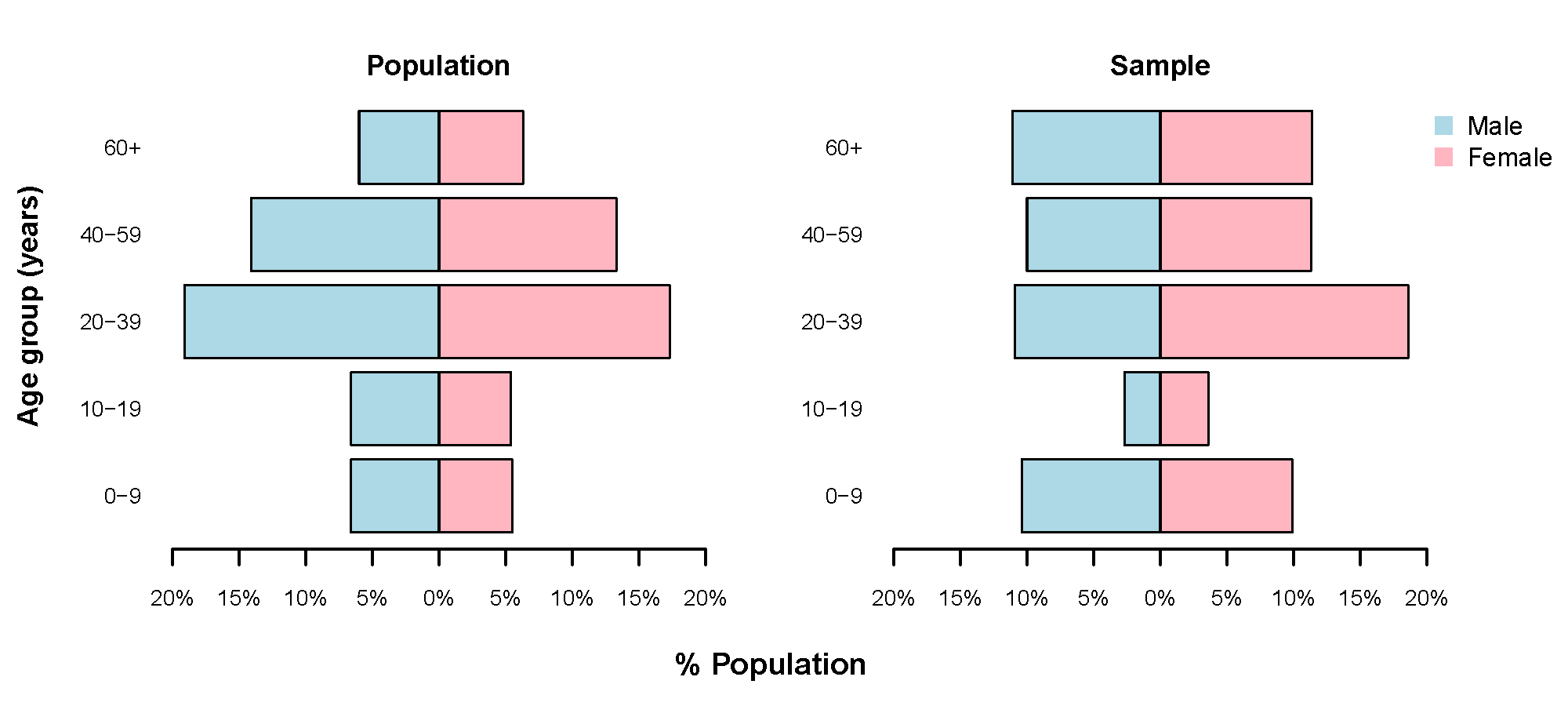

Supplement: Supplementary file 1 [file pathogens-10-01505-s001.zip › Supplemental_Figure_S1.tiff]
